# Changes in Glucose and Glutamine Lymphocyte Metabolisms Induced by Type I Interferon *α*

**DOI:** 10.1155/2010/364290

**Published:** 2010-12-27

**Authors:** Francisco Navarro, Aline V. N. Bacurau, Andréa Vanzelli, Marcela Meneguello-Coutinho, Marco C. Uchida, Milton R. Moraes, Sandro S. Almeida, Frederick Wasinski, Carlos C. Barros, Martin Würtele, Ronaldo C. Araújo, Luís F. B. Costa Rosa, Reury F. P. Bacurau

**Affiliations:** ^1^Department of Physical Education, Federal University of Maranhão, 14040-904 São Paulo, SP, Brazil; ^2^School of Physical Education and Sport, University of São Paulo, 5508-900 São Paulo, SP, Brazil; ^3^Department of Physical Education, Presbyterian University Mackenzie, 01302-907 São Paulo, SP, Brazil; ^4^Institute of Biomedical Sciences, University of São Paulo, 5508-900 São Paulo, SP, Brazil; ^5^Department of Biophysics, Federal University of São Paulo, 04023-062 São Paulo, SP, Brazil; ^6^Department of Science and Technology, Federal University of São Paulo, 12231-280 São José dos Campos, SP, Brazil; ^7^Escola de Artes, Ciências e Humanidades, Universidade de São Paulo, Avenida Arlindo Bettio, 1000, 03828-000 São Paulo, SP, Brazil

## Abstract

In lymphocytes (LY), the well-documented antiproliferative effects of IFN-*α* are associated with inhibition of protein synthesis, decreased amino acid incorporation, and cell cycle arrest. However, the effects of this cytokine on the metabolism of glucose and glutamine in these cells have not been well investigated. Thus, mesenteric and spleen LY of male Wistar rats were cultured in the presence or absence of IFN-*α*, and the changes on glucose and glutamine metabolisms were investigated. The reduced proliferation of mesenteric LY was accompanied by a reduction in glucose total consumption (35%), aerobic glucose metabolism (55%), maximal activity of glucose-6-phosphate dehydrogenase (49%), citrate synthase activity (34%), total glutamine consumption (30%), aerobic glutamine consumption (20.3%) and glutaminase activity (56%). In LY isolated from spleen, IFN*α* also reduced the proliferation and impaired metabolism. These data demonstrate that in LY, the antiproliferative effects of IFN*α* are associated with a reduction in glucose and glutamine metabolisms.

## 1. Introduction

Interferon alpha (IFN*α*) was initially characterized as an antiviral cytokine. Subsequently, several of its effects were demonstrated. Among them, the antiproliferative effect is the best characterized [1] and allows IFN*α* to be used in the treatment of several tumors [2]. IFN*α* proteins are produced both in response to infections as well as constitutively and have a wide range of functions on different cell types including the modulation of lymphocyte (LY) activity [3, 4]. Thus, this cytokine is able to modulate the proliferation, survival, and differentiation of LY [1]. The antiproliferative effect of IFN*α* on LY is related, for example, to the arrest of the cell cycle [2] and inhibition of both protein synthesis and amino acid incorporation [5]. 

LY activation is characterized by a state of high biochemical activity [6] required to sustain proliferation and the synthesis of several endogenous products in these cells [7–10]. Because in LY glucose and glutamine consumptions are strictly coupled to their cellular functions [11], the uptake and consumption of both substrates is markedly increased to cope up with the demands of activation. In this scenario, not only precursor molecules used in DNA and RNA synthesis are provided [11] but also the energy required by the biosynthetic processes [12]. Glucose and glutamine metabolisms (and consequently LY functions) can be determined by the *in vitro* measurement of some key enzymes from glycolysis, glutaminolysis, and the citric acid cycle [13]. In fact, we have previously determined the changes in LY functionality induced by different experimental conditions using this methodology [14–16]. 

Considering the antiproliferative effects of IFN*α* and the importance of the glucose and glutamine metabolisms for LY, it is tempting to speculate that IFN*α* affects the glucose and glutamine metabolisms of these cells. Thus, the aim of the present study was to evaluate the metabolism of glucose and glutamine in LY from mesenteric lymph nodes and the spleen of rats cultured in the presence of IFN*α*. Our hypothesis is that the antiproliferative effect of IFN*α* in lymphocytes can be associated to a reduction of the glucose and glutamine metabolism.

## 2. Material and Methods

### 2.1. Animals and Reagents

Male adult Wistar rats weighing 180 g (8 weeks old) from the Animal Breeding Unit, Institute of Biomedical Sciences, University of São Paulo, São Paulo, Brazil, were housed in a temperature-controlled room at 23°C under a photoperiod regimen of a 12 : 12 hrs light : dark cycle (lights on at 8:00 am) with water and commercial food *ad libitum*. These animals were maintained in accordance with the guidelines of the Brazilian Association for Laboratory Animal Science, and all experimental procedures were approved by the Ethical Committee on Animal Experimentation of the Institute of Biomedical Sciences, University of São Paulo. The [U-^14^C]-glucose, [U-^14^C]-glutamine, and [2-^14^C]-thymidine were purchased from Amersham (Little Chalfont, Buckinghamsthire, UK). All other reagents including IFN-*α* were purchased, unless specified, from Sigma (St Louis, MO, USA) or Merck (Darmstadt, Germany).

### 2.2. LY from Spleen and Mesenteric Lymph Nodes

Organs were extracted and cells extracted by pressing tissues against a steel mesh as described by Ardawi and Newsholme [17]. The cell suspension was filtered (Whatman plc, Middlesex, UK) and centrifuged at 150 g for 15 min at 4°C. The total contamination with macrophages was lower than 1%.

### 2.3. Lymphocyte Proliferation

LY from spleen and mesenteric LY were cultivated in 96-well plates (1 × 10^5^ cells per well; Corning, One Riverfront Plaza, NY, USA) under sterile conditions in GIBCO RPMI 1640 medium for 48 hrs at 37°C in an artificially humidified atmosphere of 5% CO_2_ in a microprocessor incubator (LAB LINE, Boston MA). Cells were also cultivated in the presence of concanavalin A (ConA; 5 *μ*g/mL), lipopolysaccharide (LPS; 10 mg/mL) or recombinant rat recombinant IFN*α* (1,000 U/mL; added in the beginning of culture periods). After 48 hrs in culture, more than 98% of the lymphocytes were still viable, as measured by trypan blue exclusion. The cells were labeled with 7400 Bq ^14^C-thymidine (Amersham-GE Health-care, Uppsala, Sweden) and diluted in sterile PBS yielding a final concentration of 1 *μ*g/mL. The cells were maintained under these conditions for an additional 15 hrs and automatically harvested using a multiple-cell harvester and filter paper (Skatron Combi, Sulfolk, UK). The paper discs containing the labeled cells were counted in 5 mL Bray's scintillation cocktail in a Beckman-LS 5000TD liquid scintillator (Beckman Instruments, Fullerton, CA).

### 2.4. Incubation Procedure

LY from spleen and mesenteric LY were incubated (1 × 10^6^ cells per flask) at 37°C in Krebs Ringer medium with 2% fat-free bovine serum albumin (BSA) in the presence of glucose (5 mM) or glutamine (2 mM). After 1 hr, cells were disrupted with 200 *μ*L 25% (w/v) trichloroacetic acid, and the sample was neutralized with 100 *μ*L of 0.5 M Tris containing 2.0 M KOH for the measurement of metabolites. Glucose consumption was determined as previously described by Trinder [18]. Lactate production was determined as previously described by Engle and Jones [19]. Glutamine consumption was determined using the method described by Windmueller and Spaeth [20]. All spectrophotometric measurements were performed in a Hitachi U-2001 spectrophotometer (Hitachi, Tokyo, Japan) at 25°C.

### 2.5. Glucose and Glutamine Oxidation

The ^14^CO_2_ produced from ^14^C-glucose and ^14^C-glutamine oxidation was determined as described by Curi et al. [21]. LY were incubated for 1 hr in the presence of one of the radiolabeled substrates in a sealed Erlenmeyer flask (25 mL) with one compartment for cell incubation and a second one for CO_2_ collection, as previously described by Kowalchuck et al. [22].

### 2.6. Enzymes

The activities of glucose-6-phosphate dehydrogenase (G6PDH), hexokinase (HK), and glutaminase (GLUTase), enzymes that catalyse, respectively, the first reaction of pentose phosphate and glycolitic and glutaminolytic pathways, were measured as previously described by Bergmeyer et al. [23], Crabtree and Newsholme [24], and Curthoys and Lowry [25], respectively. Citrate synthase (CS), an important enzyme from the Krebs cycle, was measured as described by Alp et al. [26]. The extraction media for enzymes were: 25 mM Tris-HCl buffer containing 1 mM EDTA and 30 mM *β*-mercaptoethanol (for HK; pH 7.4), 50 mM Tris-HCl containing 1 mM EDTA (for GLUTase: pH 8.6), 50 mM Tris-HCl containing 1 mM EDTA (for CS; pH 7.4), and 50 mM Tris-HCl containing 1 mM EDTA (for G6PDH; pH 8.0). For all enzyme assays, Triton X-100 was added to the medium to a final concentration of 0.05% (v/v). For HK activity, the following incubation medium was used (pH 7.5); 75 mM Tris-HCl containing 7.5 mM MgCl_2_, 0.8 mM EDTA, 1.5 mM KCl, 4.0 mM *β*-mercaptoethanol, 0.4 mM creatine phosphate, 1.8 U creatine kinase, 1.4 U glucose-6-posphate dehydrogenase, and 0.4 mM NADP^+^. The assay buffer for CS activity (pH 8.1) consisted of 100 mM Tris-HCl, 0.2 mM 5.5′-dithio-bis-2-nitrobenzoic acid, 15 mM acetyl-coenzyme A, and 0.5 mM oxaloacetate. The buffer for G6PDH (pH 7.6) consisted of 86 mM Tris-HCl containing 6.9 mM MgCl_2_, 0.4 mM NADP^+^, 1.2 mM glucose-6-phosphate, and 0.5% Triton X-100. The assay for GLUTase (pH 8.6) consisted of 50 mM potassium phosphate buffer containing 0.2 mM EDTA and 20 mM glutamine. In all cases, the final assay volume was 1.0 mL. CS activity was determined by absorbance at 412 nm and the other enzymes at 340 nm. All spectrophotometric measurements were performed in a Hitachi U-2001 spectrophotometer (Hitachi, Tokyo, Japan) at 25°C.

### 2.7. Protein Measurement

The protein content of the samples was measured by the method of Bradford [27]. BSA was used as standard.

### 2.8. Statistical Analysis

Analysis was performed using GraphPad-Prism. When differences among the groups were detected by two-way factorial ANOVA, the Tukey test was used. The level of significance of *P* < .05 was chosen for all statistical comparisons. Data are presented as means ± SEM.

## 3. Results

### 3.1. Lymphocytes from Mesenteric Lymph Nodes

**Figure 1:**
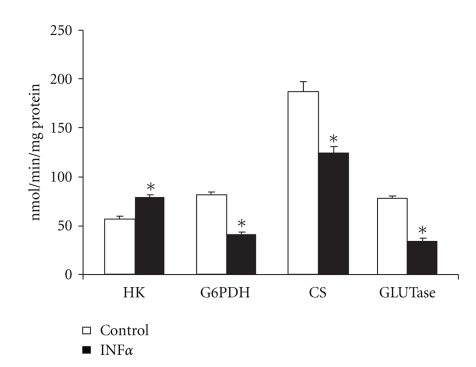
Maximal activity of enzymes of mesenteric lymphocytes cultured in the presence or absence of IFN*α*. The results are expressed as nmol/min per mg of protein and represent the mean ± SEM of 9 experiments. HK: hexokinase; G6PDH: glucose-6-phosphate dehydrogenase; CS: citrate synthase; GLUTase: phosphate dependent glutaminase. *P < .05 for comparison with the control (C) group.

**Figure 2:**
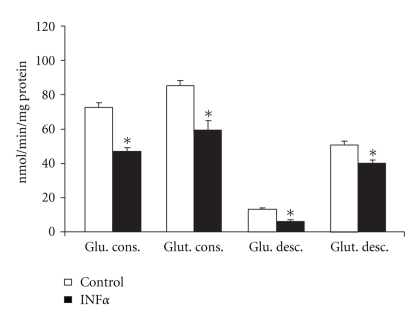
Consumption and decarboxylation of glucose and glutamine by mesenteric lymphocytes cultured in the presence or absence of IFN*α*. The results are expressed as nmol/min per mg of protein and represent the mean ± SEM of 9 experiments. *P < .05 for comparison with the control (C) group.

Lymphocytes obtained from mesenteric lymph nodes cultured in the presence of IFN*α* (1000 U/mL for 48 hrs) presented a reduced proliferative index under all evaluated conditions when compared to cells cultivated without this cytokine (reduction by 13%, 24.4%, and 33.5%, when compared to control, concanavalin A, and LPS experiments, respectively) (Table 1). This reduction was accompanied by a reduction of 49.2% of the maximal activity of glucose-6-phosphate dehydrogenase (G6PDH) (Figure 1). Glucose utilization for energetic processes was also reduced by IFN*α* as can be seen by a 35.3% reduction in glucose consumption and a 55% decrease in glucose decarboxylation (Figure 2). On the other hand, maximal activity of hexokinase (HK) increased by 1.4-fold in cells incubated with IFN*α* (Figure 1). The maximal activities of citrate synthase (CS) and glutaminase (GLUTase assay) were also reduced in lymphocytes incubated in the presence of IFN*α* when compared to cells incubated without the cytokine (34% and 56% reduction, resp.) (Figure 1). In agreement with the result of the GLUTase assay, glutamine consumption (−30.2%) and glutamine aerobic utilization (−20.3%) were reduced by IFN*α* in comparison to cells incubated without the cytokine (Figure 2).

### 3.2. Lymphocytes from Spleen

**Figure 3:**
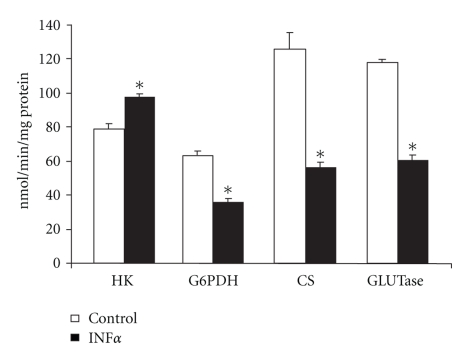
Maximal activity of enzymes of lymphocytes from spleen cultured in the presence or absence of IFN*α*. The results are expressed as nmol/min per mg of protein and represent the mean ± SEM of 9 experiments. HK: hexokinase; G6PDH: glucose-6-phosphate dehydrogenase; CS: citrate synthase; GLUTase: phosphate dependent glutaminase. *P < .05 for comparison with the control (C) group.

**Figure 4:**
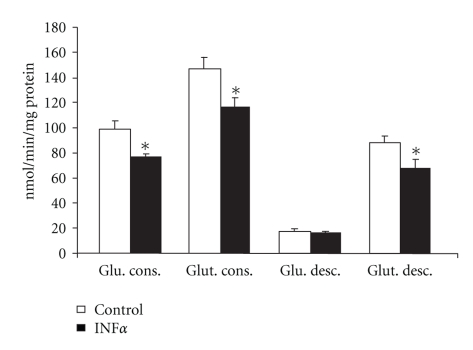
Consumption and decarboxylation of glucose and glutamine by lymphocytes from spleen cultured in the presence or absence of IFN*α*. The results are expressed as nmol/min per mg of protein and represent the mean ± SEM of 9 experiments. *P < .05 for comparison with the control (C) group.

In lymphocytes obtained from the spleen, IFN*α* promoted the same pattern of changes in glucose and glutamine metabolism observed in lymphocytes from mesenteric lymph nodes. In comparison to control cells cultivated without IFN*α*, lymphocytes from the spleen presented a reduced proliferative index in all conditions evaluated (reduction by 31.3%, 33.1%, and 27%, when compared with control, concanavalin A, and LPS experiments, resp.) (Table 1). As observed for lymphocytes obtained from mesenteric lymph nodes, most of the features of glucose metabolism in LY from the spleen were reduced by IFN*α*, as can be seen by the reduction of 43% in maximal G6PDH activity (Figure 3) and a reduction of 22% in glucose consumption (Figure 4). Again, the exception in glucose metabolism was the 1.2-fold increased maximal HK activity observed in the spleen LY when they were incubated in the presence of IFN*α* in comparison to control cells (Figure 3). Glutamine metabolism, on the other hand, was also reduced in these LY due to IFN*α* activity. Glutamine consumption decreased 21% and glutamine decarboxylation was reduced 23% in the presence of IFN*α* in comparison to control cells (Figure 4). Glutamine decarboxylation was accompanied by a reduction of 55.3% of the activity of important enzymes from the citric acid cycle (Figure 3).

## 4. Discussion

The antiproliferative effect of IFN*α* has been well described in different cell types [2] and has been related to the ability of these cytokines to affect several processes (e.g., protein synthesis) required for LY activation [5]. Herein, we demonstrate that glucose and glutamine metabolisms, particularly important for LY activation [17], are also modulated by IFN*α*. 

As expected, our results confirm the antiproliferative effect of IFN*α* on LY from mesenteric lymph nodes and the spleen. In fact, in a general sense, the cytokine promoted the same pattern of changes in the metabolism of LY from these diverse locations. Hence, the data of both LY populations will be discussed together.

Confirming the strict relation between substrate use and function in LY [11], the antiproliferative effect of IFN*α* was accompanied by a reduction in glucose and glutamine metabolisms. Thus, our results added the reduction of both substrates to the list of known factors related to the antiproliferative effect of IFN*α*. 

In spite of being a nonessential amino acid, several conditions such as infection and injuries can lead glutamine to become “conditionally essential”. From this perspective, investigations about the rate of utilization of glutamine by immune cells have been performed aiming to open new ways of therapeutic manipulation of the proliferative, phagocytic, and secretory capacities of these cells [11]. As an example, the lymphocyte mitogen concanavalin A increased both glutaminase activity as well as glutamine utilization [28]. In this study, the antiproliferative effect of IFN*α* on LY was, however, accompanied by a reduction in glutaminase maximal activity and glutamine consumption. Furthermore, reductions of citrate synthase (CS) activity and of glutamine decarboxylation demonstrate that aerobic pathways linked to the metabolism of this amino acid were also affected by IFN*α*.

Although both glucose and glutamine are utilized for energy production by LY, the first seems to be quantitatively more important [12]. In this study, LY cultured in the presence of IFN*α* consumed less total glucose and presented a reduced metabolism of this substrate by aerobic pathways as demonstrated by the minor glucose decarboxylation and activity of CS. Besides energy production, the reduction of the maximal activity of G6PDh, the first enzyme of the pentose-phosphate pathway, suggests that IFN*α* also compromises proliferation by reducing the production of metabolites and precursors needed for the biosynthesis of cell components essential for proliferation [29]. Still considering glucose metabolism, it is interesting to note that in spite of the reduced glucose consumption, IFN*α* increased the maximal activity of HK suggesting that the conversion of glucose to glucose-6-phosphate was not affected by this cytokine. Upon activation, LY increase their glucose uptake via GLUT1 [30]. Thus, even if the increased HK activity represents a greater glucose uptake in cultured LY the greater enzyme activity was not enough to promote an augment in glucose consumption because the subsequent processes of glucose metabolism were downregulated by IFN*α*.

In accordance with MacIver et al. [30], the understanding of how normal LY function is regulated and fueled to allow production of ATP and biosynthetic precursors essential for growth and the effector function of these cells is very important due the severe downregulation of immune functions which result from LY deficiencies. 

Additionally, because many cancer cells consume glucose in a manner similar to LY, that is, converting glucose to lactate even in the presence of enough oxygen [31], it is tempting to speculate that the results of the present study could be relevant for the understanding of the role of IFN*α* as an anticancer agent. Supporting this speculation, it has been demonstrated that different cancer cells can be resistant to IFN*α* [32, 33]. This effect could be associated with an inadequate activation of the JAK-STAT pathway and its effectors STAT1 and STAT2. In this scenario, while adequate levels of STAT1 are pivotal for the establishment of IFN*α* effects, low levels or overexpression of this transcription factor seems to be advantageous for tumor cells [32]. Interestingly, a previously uncharacterized role of STAT1 in regulating the expression of genes involved in glycolysis, citrate cycle, and oxidative phosphorylation has been recently demonstrated. On the other hand, we previously were able to demonstrate that LY of tumor-bearing rats presented reduced proliferation, glucose consumption, and maximal activity of enzymes such as G6PDH and CS, while simultaneously, Walker 256 tumor cells of the same animals presented an increased glucose metabolism [34].

As IFN*α* has antiapoptotic effects on activated LY [35] which are modulated by the metabolism of glucose and glutamine [15], the metabolism of these substrates and LY proliferation can be correlated with collagen-induced arthritis [16], and the high glucose and lipid levels observed in individuals with type 2 diabetes and obesity contribute to LY activity promoting inflammation [30]. The results presented here could be of relevance to other fields related with immunology. 

Thus, further investigations concerning the molecular mechanisms underlying the effects of IFN*α* (and other cytokines) upon glucose and glutamine metabolisms as well as proliferation of LY could lead to the development of strategies to target cancer, autoimmune diseases and chronic diseases.

## 5. Conclusions

In conclusion, our data suggest that the inhibition of glucose and glutamine metabolism is an important part of the mechanism of the antiproliferative effect of IFN*α* in lymphocytes from rats.

## Figures and Tables

**Table 1:** Proliferation of splenocytes and mesenteric lymphocytes cultured in the presence or absence of IFN*α*.

	No add	ConA	LPS
C LFN	1003.6 ± 65.3	1954.5 ± 87.5	1753.1 ± 103.2
IFN LFN	875.4 ± 65.8**^†^**	1478.3 ± 76.3**^†^**	1165.9 ± 55.9**^†^**
C SPL	1231.2 ± 81.9	2309.6 ± 117.4	1987.3 ± 80.2
IFN SPL	845.1 ± 76.4^♦^	1543.9 ± 67.1^♦^	1456.3 ± 87.3^♦^

The values are expressed as disintegrations per minute (DPM) and are presented as mean ± SEM of 9 experiments. ConA: concanavalin A; LPS: lipopolysaccharide; C LFN: mesenteric lymphocytes incubated in the absence of IFN*α*; IFN LFN; mesenteric lymphocytes cultured with IFN*α*; C SPL: splenocytes cultured in the absence of IFN*α*; SPL IFN splenocytes cultured with IFN*α*. **^†^** *P* < .05 compared to C LY group. ^♦^ *P* < .05 compared with C SPL group.
